# Effect of Oral Glucose Administration on Rebound Growth Hormone Release in Normal and Obese Women: The Role of Adiposity, Insulin Sensitivity and Ghrelin

**DOI:** 10.1371/journal.pone.0121087

**Published:** 2015-03-17

**Authors:** Lara Pena-Bello, Sonia Pertega-Diaz, Elena Outeiriño-Blanco, Jesus Garcia-Buela, Sulay Tovar, Susana Sangiao-Alvarellos, Carlos Dieguez, Fernando Cordido

**Affiliations:** 1 Department of Medicine, Faculty of Health Sciences, University of A Coruña, A Coruña, Spain; 2 Instituto de Investigación Biomedica (INIBIC), University Hospital A Coruña, A Coruña, Spain; 3 Clinical Epidemiology and Biostatistics Unit, University Hospital A Coruña, A Coruña, Spain; 4 Department of Endocrinology, University Hospital A Coruña, A Coruña, Spain; 5 Department of Physiology (CIMUS), School of Medicine-Instituto de Investigaciones Sanitarias (IDIS), Universidad de Santiago de Compostela, Santiago de Compostela, Spain, and CIBER Fisiopatología de la Obesidad y Nutrición (CIBERobn), Santiago de Compostela, Spain; Complexo Hospitalario Universitario de Santiago, SPAIN

## Abstract

**Context:**

Metabolic substrates and nutritional status play a major role in growth hormone (GH) secretion. Uncovering the mechanisms involved in GH secretion following oral glucose (OG) administration in normal and obese patients is a pending issue.

**Objective:**

The aim of this study was to investigate GH after OG in relation with adiposity, insulin secretion and action, and ghrelin secretion in obese and healthy women, to further elucidate the mechanism of GH secretion after OG and the altered GH secretion in obesity.

**Participants and Methods:**

We included 64 healthy and obese women. After an overnight fast, 75 g of OG were administered; GH, glucose, insulin and ghrelin were obtained during 300 minutes. Insulin secretion and action indices and the area under the curve (AUC) were calculated for GH, glucose, insulin and ghrelin. Univariate and multivariate linear regression analyses were employed.

**Results:**

The AUC of GH (μg/L•min) was lower in obese (249.8±41.8) than in healthy women (490.4±74.6), P=0.001. The AUC of total ghrelin (pg/mL•min) was lower in obese (240995.5±11094.2) than in healthy women (340797.5±37757.5), P=0.042. There were significant correlations between GH secretion and the different adiposity, insulin secretion and action, and ghrelin secretion indices. After multivariate analysis only ghrelin AUC remained a significant predictor for fasting and peak GH.

## Introduction

Adiposity is associated with decreased growth hormone (GH) secretion [1]. The altered somatotroph function of obesity is not permanent; it can be reversed by a return to normal weight [1]. The most striking secretory capacity appeared when obese subjects were treated with GH-releasing hormone (GHRH) plus GH-Releasing Peptide-6, which resulted in a massive GH response for obese subjects [2]. Clinical trials assessing the effects of GH treatment in patients with obesity have shown reductions in total adipose tissue mass, especially abdominal and visceral adipose tissue depots [3]. In animals, an exacerbation of the age-associated decline in pulsatile GH secretion has been found in high-fat-fed mice, as well as dietary-induced weight gain [4].

The mechanism of altered GH secretion in obesity is unclear. Insulin has been shown to reduce GH secretion in the animal model [5]. In humans, low-level insulin infusions, has been found to reduce the GH response to GHRH in a dose-dependent manner [6]. Fasting insulin and abdominal visceral fat are important predictors of integrated 24-h GH concentrations in healthy adults [7]. Cornford et al found that overeating induced a rapid and sustained suppression of GH secretion. The reduction in GH secretion occurred before any change in body mass and the markedly decreased GH secretion was accompanied by an increase in plasma insulin concentration [8]. There is strong evidence that ghrelin stimulates appetite and increases circulating GH across varied patient populations [9]. Studies to determine the effects of endogenous ghrelin on the control of GH secretion have yielded conflicting results. Avram et al. [10] did not observe any relationship with GH under fed or fasting conditions. Koutkia et al. [11] found that there is a significant regularity in cosecretion between ghrelin and GH in the fasting state. Misra et al. [12] found that fasting ghrelin is an independent predictor of basal GH secretion and GH secretory burst frequency. Nass et al [13] found that under normal conditions in subjects given regular meals, endogenous acylated ghrelin acts to increase the amplitude of GH pulses. Ghrelin secretion is decreased in obesity [14] and could be responsible for altered GH secretion in obesity.

The oral glucose tolerance test is a clinical model to study the different indices of insulin secretion and action [15, 16], and is also an excellent stimulus for evaluating ghrelin [17] and GH secretion. There is evidence that oral glucose (OG) administration affects GH secretion, initially decreasing and subsequently stimulating GH secretion and in human obesity GH secretion after OG is decreased [18]. With a single test we can study insulin, GH and ghrelin secretion. Circulating plasma ghrelin increases before a meal and decreases following the consumption of nutrients and after OG [17]. Insulin rises after OG and has been suggested to decrease circulating ghrelin levels [19]. Enhanced acylated ghrelin suppression persists for up to 2 years after Roux-en-Y gastric bypass, and this effect is associated with decreased android obesity and improved insulin secretion [20]. Ghrelin infused to levels occurring in physiologic states such as starvation decreases insulin secretion [21]. Therefore, a possible role for insulin as a common regulator of circulating ghrelin and GH after OG cannot be excluded. In preliminary studies in a small number obese and healthy women, a significant correlations between post-oral glucose GH secretion and ghrelin secretion have been found [22], although the putative contribution of other factors like insulin secretion and action indices or leptin were not studied in depth.

We hypothesized that among the variables known to regulate GH secretion in obesity, it would be possible to determine the relative importance of the predictors that contribute to GH secretion after oral glucose in women. Our aim was to study fasting GH concentrations and their response to OG administration in relation with adiposity, insulin secretion and action indices and ghrelin secretion in obese and healthy women, in order to elucidate the hypothetical mechanism of GH secretion after OG and the altered GH secretion in obesity.

## Patients and Methods

### Patients

All the studies have been conducted in accordance with the Declaration of Helsinki. The study protocol was approved by our centre′s ethical committee (Hospital A Coruña, Xunta de Galicia), and written informed consent was obtained from all patients and controls. We included a total of sixty-four women in our study. Forty obese women, aged 38.9±2.0 yrs., with a body mass index (BMI) of 37.7±1.0 kg/m^2^, were studied. As a control group, we studied twenty-four healthy women, selected from a pool of volunteers available to our unit, aged 37.1±2.4 yrs., and with a BMI of 22.9±0.5 kg/m^2^ (selected in a 2:1 ratio). Both groups were homogeneous and only differed in terms of their BMI. None of the obese patients or controls had diabetes mellitus or other medical problems, nor were they taking any drugs. The subjects had been eating a weight-maintaining diet for several weeks prior to the study. We specifically tell the patients that they should maintain their usual eating and exercise habits during the previous two weeks of the study.

### Study procedure

Between 08.30 and 09.00 a.m., after an overnight fast and while seated, a peripheral venous line was obtained. Fifteen minutes later 75 g of oral glucose were administered. All of the studies were done during the first ten days from the beginning of the menstrual period. We obtained blood samples for glucose, insulin, GH and ghrelin at baseline (fasting) and then at 30, 60, 90, 120, 150, 180, 210, 240, 270 and 300 minutes. Basal levels of leptin and insulin-like growth factor 1(IGF-1) were also measured. All blood samples were immediately centrifuged, separated and frozen at -80°C. Samples destined to be used for the determination of plasma ghrelin were specifically retrieved in chilled tubes containing aprotinin and EDTA-Na, and then immediately centrifuged at 4°C., separated to aliquots and frozen at -80°C. Mid-waist circumference was measured as the midpoint between the iliac crest and the lowest rib, with the patient in the upright position. Total body fat was calculated through bioelectrical impedance analysis (BIA). The independent variables examined included: age, gender, BMI, body fat, waist circumference, fasting glucose, fasting insulin, HOMA-IR HOMA-β, Matsuda index, Glucose area under the secretory curve (AUC), Insulin AUC, Insulin/Glucose AUC, fasting Ghrelin, ghrelin AUC.

### Assays and other methods

Serum samples were collected and stored at-80 C. Serum GH (μg/L) was measured by a solid-phase, two-site chemiluminescent enzyme immunometric assay (Immulite, EURO/DPC) with a sensitivity of 0.01 μg/L and with intra-assay coefficients of variation of 5.3%, 6.0% and 6.5% for low, medium and high plasma GH levels respectively; and with inter-assay coefficients of variation of 6.5%, 5.5% and 6.6% for low, medium and high plasma GH levels respectively. IGF-1 (ng/mL) was determined by a chemiluminescence assay (Nichols Institute, San Clemente, CA, USA) and with intra-assay coefficients of variation of 4.8%, 5.2% and 4.4% for low, medium and high plasma IGF-1 levels respectively; and with interassay coefficients of variation of 7.7%, 7.4% and 4.7% for low, medium and high plasma IGF-I levels respectively. Insulin (μU/mL) was measured with a solid-phase two-site chemiluminescent mmunometric assay (Immulite 2000 Insulin, DPC, Los Angeles, CA, USA) and with intra-assay coefficients of variation of 5.5%, 3.3% and 3.7% for low, medium and high plasma insulin levels respectively; and with inter-assay coefficients of variation of 7.3%, 4.1% and 5.3% for low, medium and high plasma insulin levels respectively. Leptin (ng/mL) was measured by radioimmunoassay (Mediagnost, Tubigen, Germany) and with intra-asay and inter-assay coefficients of variation of 5.3% and 13.6% respectively. Total ghrelin (pg/ml) was measured by a commercially available radioimmunoassay (RIA) (Linco Research Inc., St Charles, MO, USA), specific for total ghrelin, that uses ^125^I-labeled ghrelin tracer and rabbit antighrelin serum with a specificity of 100%, with an intra-assay coefficient of variation between 3.3–10% and an inter-assay coefficient of variation between 14.7–17.8. Plasma glucose (mg/dL) was measured with an automatic glucose oxidase method (Roche Diagnostics, Mannheim, Germany). All samples from a given subject were analysed in the same assay run.

### Calculations

The area under the secretory curve (AUC) was calculated with the trapezoidal rule (0–300 minutes).

Insulin sensitivity (IS) was measured with the following methods: HOMA-IR with the formula: fasting serum insulin (μU/mL) x fasting plasma glucose (mmol/L)/22.5; quantitative IS check index (QUICKI) with the formula: 1/[log insulin(μU/mL) + log glucose (mg/dL)]; Matsuda index with the formula: 10,000/√(fasting plasma glucose (mmol/L) x fasting plasma insulin (μU/mL)) x (mean plasma glucose_0–120_ x mean plasma insulin_0–120_). For HOMA-IR, lower values indicate higher IS; for QUICKI and Matsuda index, higher values indicate higher IS [15, 16].

Insulin secretion was estimated using the basal insulin values by the HOMA-β: [20 x fasting insulin (μU/mL)]/[fasting plasma glucose (mmol/L)– 3.5]. Total insulin secretion after oral glucose was calculated as the AUC after oral glucose and total glucose-adjusted insulin response after oral glucose using the ratios of the areas of the insulin and glucose curves (AUC I/G) [15, 16].

### Statistical analysis

Quantitative variables were expressed as mean (standard error) and median. Mann-Whitney test was used to compare obese and control women with respect to biochemical data, hormonal records and insulin secretion and action indices.

Association between GH secretion indices and the different adiposity, insulin secretion, insulin action and ghrelin secretion indices was analyzed by means of Spearman’s Rho correlation coefficient. The linearity of associations with GH secretion indices were additionally explored by means of penalized cubic regression splines.

In the multivariate analysis, both linear regression and generalized additive (GAM) models were used to investigate the variables associated with different GH secretion indices. In both regression analyses, fasting GH, peak GH and GH area under the curve were log-transformed because of skewness. Mathematically, in GAM models some covariates can be replaced by arbitrary smooth functions, so finally they were fitted to allow for nonlinear effects detected in some of the variables studied.

Statistical analysis was carried out using the R 2.15.1 software (R Foundation for Statistical Computing, Vienna, Austria) and the Statistical Package for Social Sciences version 19.0 for Windows (IBM, Armonk, NY, USA). All statistical tests were two-sided. Only p-values <0.05 were considered as statistically significant.

## Results

The age and adiposity indices (mean±SE) of the healthy control group and patients are shown in Table 1. Waist circumference (cm) was higher in the obese women than in healthy control women; 108.8±2.1 vs 81.8±1.4 p<0.001, for the obese patients and control respectively.

**Table 1:** Age, adiposity indices, fasting and after oral glucose biochemical and hormonal data, and insulin secretion and action indices (mean±SE) in obese patients and healthy control women.

	Controln = 24		Obesen = 40		
	Mean(SE)	Median	Mean(SE)	Median	p
**Age (years)**	37.1 (2.4)	34.5	38.9 (2.0)	39.5	0.390
**Body mass index (kg/m2)**	22.9 (0.5)	22.8	37.7 (1.0)	36.2	<0.001
**Waist (cm)**	81.8 (1.4)	80.5	108.8 (2.1)	109.0	<0.001
**Body fat mass (kg)**	20.4 (1.3)	19.1	44.4 (2.0)	41.5	<0.001
**Body fat (%)**	30.3 (0.8)	29.2	45.3 (0.7)	45.1	<0.001
**Fasting Glucose (mg/dL)**	90.0 (2.0)	92.0	101.7 (2.7)	97.0	0.002
**Fasting Insulin (**μ**UI/mL)**	5.3 (1.0)	4.3	15.1 (4.2)	10.6	<0.001
**Fasting GH (**μ**g/L)**	2.1 (0.6)	0.7	0.9 (0.2)	0.5	0.152
**Fasting IGF-1 (ng/mL)**	159.5 (8.7)	160.5	130.0 (9.2)	120.5	0.010
**Fasting Leptin (ng/mL)**	22.8 (2.2)	20.3	67.3 (4.5)	67.3	<0.001
**Fasting Ghrelin (pg/mL)**	1317.6 (163.9)	1054.5	889.4 (45.5)	883.5	0.026
**Peak GH (**μ**g/L)**	6.6 (0.8)	4.9	3.3 (0.5)	2.2	<0.001
**AUC**_**0–300**_ **GH (**μ**g/L·min)**	490.4 (74.6)	373.3	249.8 (41.8)	166.9	0.001
**Peak Glucose (mg/dL)**	153.2 (7.3)	149.0	174.7 (8.6)	160.5	0.155
**AUC**_**0–300**_ **Glucose (mg/dL·min)**	28718.7 (1072.0)	29370.0	33794.2 (1574.8)	31725.0	0.005
**Peak Insulin (**μ**UI/mL)**	61.3 (8.4)	49.7	100.7 (9.3)	80.6	0.001
**AUC**_**0–300**_ **Insulin (**μ**UI/mL·min)**	7668.2 (1125.6)	6385	12392.6 (947.9)	11173.0	<0.001
**HOMA-β**	67.4 (8.9)	67.5	111.6 (10.6)	103.9	0.008
**AUC**_**0–300**_ **Insulin/glucose (**μ**UI/mL)/ (mg/dL)·min**	0.23 (0.02)	0.2	0.4 (0.03)	0.3	<0.001
**HOMAir**	1.2 (0.2)	0.8	2.9 (0.3)	2.5	<0.001
**QUICKI**	0.4 (0.01)	0.4	0.3 (0.01)	0.3	<0.001
**Matsuda Index**	13.1 (1.1)	12.4	5.3 (0.4)	4.8	<0.001
**Nadir ghrelin (pg/mL)**	886.1 (100.1)	756.0	660.0 (35.4)	684.0	0.124
**AUC**_**0–300**_ **ghrelin (pg/mL·min)**	340797.5 (37757.5)	280372.5	240995.5 (11094.2)	242325.0	0.042

*SE*: *Standard Error*, *AUC*_*0–300*_, *area under the secretory curve between time 0–300 min*

### Fasting serum levels

The fasting serum levels (mean±SE) are presented in Table 1.

Fasting total ghrelin (pg/mL) levels were lower in obese women than in healthy control women; 889.4±45.5 vs 1317.6±163.9, p = 0.026, for the obese and healthy women respectively. Fasting IGF-1 (ng/mL) levels were lower in obese women than in healthy control women; 130.0±9.2 vs 159.5±8.7, p = 0.01, for obese and healthy women respectively.

#### Serum levels after oral glucose

**Fig 1:**
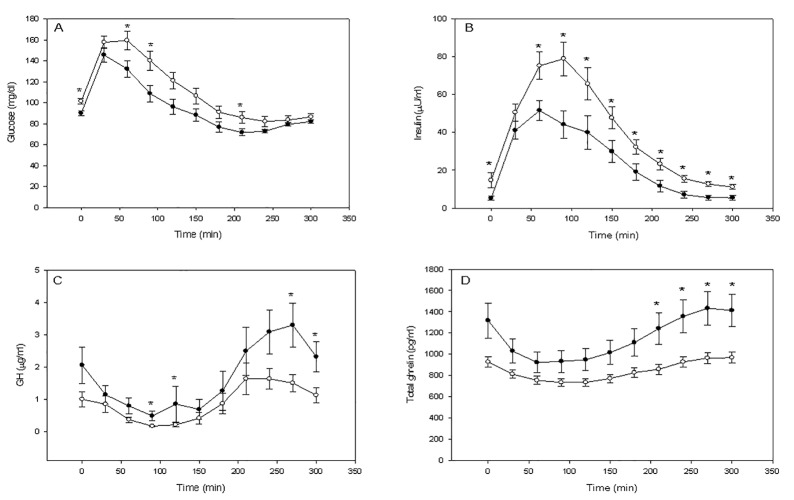
Mean±SE plasma glucose (a; mg/dL), insulin (b; μU/mL) GH (c; μg/L) and total ghrelin levels (d; pg/mL) in normal (grey) and obese women (black) during the prolongued oral glucose tolerance test. * p<0.05 between normal and obese women at that time point. Insulin was higher in the obese women than in the healthy control women after the 300 min OG (Fig. 1b). GH was lower in the the obese women than in the healthy control women after OG (Fig. 1c). The AUC of GH (μg/L·min) between 0 and 300 min was lower in the obese patients than in the controls; 249.8±41.8 vs 490.4±74.6, P = 0.001, for the obese patients and controls respectively. Peak GH (μg/L) levels were lower in the obese than in the healthy women, 3.3±0.5 vs 6.6±0.8 p<0.001, for the obese and healthy women respectively. Total ghrelin was lower in the obese women than in the healthy women after OG (Fig. 1d). The AUC of total ghrelin (pg/mL·min) between 0 and 300 min was lower in the obese patients than in the controls; 240995.5±11094.2 vs 340797.5±37757.5, P = 0.042, for the obese and healthy women respectively (Fig. 2).

The post-oral glucose serum levels (mean±SE) are presented in Table 1. Glucose was higher in the obese women than in the healthy control women after the 300 min OG (Fig. 1a).

**Fig 2:**
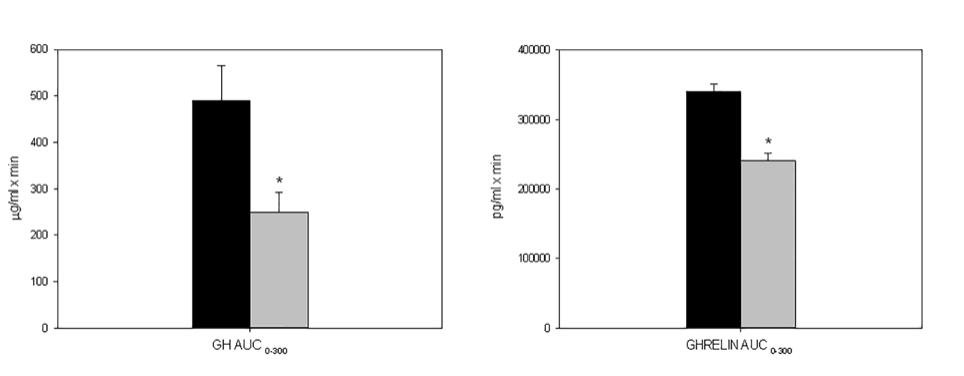
Mean±SE plasma GH AUC_0–300_ (pg/mL·min) and Ghrelin AUC_0–300_ (pg/mL·min) in normal (grey) and obese women (black) during the prolongued oral glucose tolerance test. * p<0.05 between normal and obese women.

### Insulin secretion and action indices

The insulin secretion and action indices (mean±SE) are presented in Table 1. HOMA-β levels were higher in obese women than in healthy women; 111.6±10.6 vs 67.4±8.9, p = 008, for the obese and healthy women respectively. AUC_0–300_ Insulin/glucose ((μU/mL)/(mg/dL)·min) levels were higher in the obese women than in the healthy control women; 0.4±0.03 vs 0.23±0.02, P<0.001, for the obese and healthy women respectively. HOMA-IR levels were higher in the obese women than in the healthy control women; 2.9±0.3 vs 1.2±0.2, p<0.001, for the obese and healthy women respectively. Matsuda index was lower in the obese women than in the healthy control women; 5.3±0.4 vs 13.1±1.1, P<0.001, for the obese and healthy women respectively.

### Correlations

There were significant association between GH secretion indices and the different adiposity, insulin secretion, insulin action and ghrelin secretion indices (Table 2).

**Table 2:** Correlations between GH secretion indices (Peak: μg/L and AUC: μg/L·min) and the different adiposity indices, insulin secretion indices, insulin action indices and ghrelin (Nadir: pg/mL and AUC: pg/mL·min) secretion in the entire group of healthy and obese women.

	GH Fasting		GH Peak		GH AUC_0–300_	
	Rho	p	Rho	p	Rho	p
**IGF-1**	-0.062	0.635	0.279	0.028	0.323	0.010
**BMI**	-0.214	0.095	-0.564	<0.001	-0.499	<0.001
**Waist circumference**	-0.197	0.124	-0.560	<0.001	-0.465	<0.001
**Total body Fat (%)**	-0.227	0.076	-0.479	<0.001	-0.414	0.001
**Total body Fat mass**	-0.241	0.059	-0.561	<0.001	-0.500	<0.001
**Leptin**	-0.218	0.094	-0.425	0.001	-0.419	0.001
**Fasting glucose**	-0.287	0.024	-0.582	<0.001	-0.552	<0.001
**Fasting Insulin**	-0.248	0.052	-0.482	<0.001	-0.511	<0.001
**Homa-β**	-0.160	0.214	-0.220	0.086	-0.268	0.035
**AUC Insulin**_**0–300**_	-0.155	0.228	-0.408	0.001	-0.449	<0.001
**AUC Insulin/Glucose**_**0–300**_	-0.115	0.373	-0.245	0.055	-0.294	0.020
**Homa-IR**	-0.252	0.059	-0.540	<0.001	-0.575	<0.001
**QUICKI**	0.242	0.059	0.523	<0.001	0.552	<0.001
**Matsuda Index**	0.243	0.057	0.553	<0.001	0.559	<0.001
**Disposition index**	0.213	0.097	0.468	<0.001	0.431	<0.001
**Fasting Ghrelin**	0.386	0.002	0.450	<0.001	0.443	<0.001
**Nadir Ghrelin**	0.401	0.001	0.341	0.007	0.371	0.003
Ghrelin AUC_0–300_	0.001	0.412	0.001	0.409	0.001	0.399

*Rho*: *Spearman’s Rho correlation coefficient; AUC*_*0–300*_, *area under the secretory curve between time 0–300 min*.

**Fig 3:**
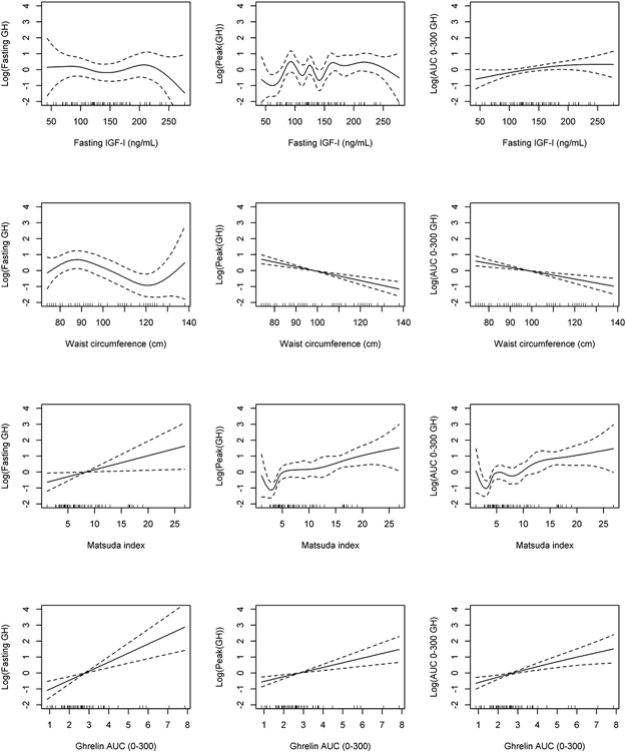
Relationship between GH secretion indices (log-transformed) and IGF-I, waist circumference, Matsuda index and Ghrelin AUC_0–300_ data. **Univariate generalized additive regression (GAM) models.** A clear linear association between ghrelin AUC and the different indices of GH secretion (log-transformed) was found (Fig. 3).

The linearity of associations with GH secretion indices were additionally explored by means of penalized cubic regression splines (Fig. 3).

Due to the significant correlations between the different adiposity indices, insulin secretion indices, insulin action indices and ghrelin, multivariate analysis was used with both linear regression and generalized additive (GAM) models in order to quantitatively assess the individual contribution that each predictive measure made to explain the variability among the values of GH secretion indices (fasting, peak, AUC) while in the presence of the remaining predictors. In the presence of IGF-I, adiposity indices (waist circumference), insulin sensitivity (Matsuda index) and ghrelin secretion (ghrelin AUC/100.000), only ghrelin AUC remained a significant predictor for fasting GH and Peak GH, and was borderline significant for GH AUC (Table 3).

**Table 3:** Generalized additive regression (GAM) model for GH secretion indices (log-transformed).

	**Fasting GH (log(**μ**g/L))**			**Peak GH (log(**μ**g/L))**			**AUC**_**0–300**_ **GH (log(**μ**UI/mL·min))**		
	**B**	**SE**	**p**	**B**	**SE**	**p**	**B**	**SE**	**p**
**Intercept**	-3.266	0.896	<0.001	2.398	0.846	0.007	5.871	0.897	<0.001
**Age (years)**	0.011	0.017	0.537	-0.012	0.008	0.148	-0.009	0.008	0.280
**IGF-1** *	-	-	0.993	-	-	0.231	-	-	0.138
**Waist circumference** *	-	-	0.149	-0.012	0.007	0.097	-0.005	0.008	0.491
**Matsuda Index**	0.098	0.055	0.080	-	-	0.150	-	-	0.139
**GhrelinAUC**_**0–300**_ **(/100.000)**	0.430	0.160	**0.010**	0.159	0.077	**0.045**	0.162	0.081	0.053

*Introduced as a non-linear term in the model

*AUC*_*0–300*_, *area under the secretory curve between time 0–300 min; B*, *linear regression coefficient; SE*, *standard error*

## Discussion

By studying a relative large cohort of women, we have found that after OG in healthy and obese women, both ghrelin and GH secretion are decreased. There was a significant association between the GH secretion indices and the different adiposity, insulin secretion, insulin action indices and ghrelin secretion. After multivariate analysis, only ghrelin secretion remained a significant predictor for both fasting and peak GH. These data suggest that circulating ghrelin is an important regulator of GH secretion after OG in women, and that the decreased GH secretion in obesity after OG is probably due to the altered ghrelin secretion found in obese women. Taken together, these data further reinforces the importance of metabolic signals and nutritional status in GH secretion which in turn could influence the perpetuation and/or further development of the obese state.

The primary cause of impaired GH secretion in obesity could be an altered hypothalamus, abnormal pituitary function, or a perturbation of the peripheral signals acting at either the pituitary or hypothalamic level. There are three important peripheral signals that could participate in the altered GH secretion of obesity, leptin, insulin and ghrelin. Iranmanesh et al studied GH secretion during 6 h. after OG in men [23]. They found that glucose-suppressed nadir GH concentrations and postglucose rebound-like peak GH release in men are strongly determined by selective metabolic surrogates, especially including adipose visceral fat, adiponectin, leptin and sex hormone binding globulin (SHBG). Our data are in accordance with their suggestion that the most likely mechanism of rebound GH secretion after glucose ingestion is delayed endogenous ghrelin drive under waining somatostatin restraint. **T**he best established action of exogenously administered ghrelin is its potent stimulation of pituitary GH secretion [9]. In humans a *GHSR* missense mutation, which impairs the constitutive activity of the GHS-R, is associated with short stature [24]. Several studies have investigated the relationship between ghrelin and GH secretion. Espelund et al. [25], did not find a correlation between ghrelin and GH under fasting conditions. Avram et al. [10] did not observe any relationship with GH under fed or fasting conditions. Their differing results may be connected with protocol differences, and also their study population included both men and women. Koutkia et al. [11] found that there is a significant regularity in cosecretion between ghrelin and GH in the fasted state; however, this was only found during the night. Misra et al. [12], using deconvolution analysis for GH and total ghrelin in healthy adolescents and adolescents with anorexia, found that fasting ghrelin is an independent predictor of basal GH secretion and GH secretory burst frequency. In studies carried out on 8 healthy young men, Nass et al [13] found a significant relationship between GH secretion peak amplitudes and mean circulating acylated ghrelin levels during the fed condition. In agreement with our results in healthy and obese women, they conclude that under normal conditions in subjects given regular meals, endogenous ghrelin acts to increase the amplitude of GH pulses. Studies in mice without the Ghrelin O-acyltransferase gene (*Mgoat*) have found that an essential function of ghrelin in mice is elevation of GH levels during severe calorie restriction, thereby preserving blood glucose and preventing death [26]. On the other hand, it has been recently found that with normal aging, endogenous acyl-ghrelin levels are less tightly linked to GH regulation [27], indicating that the physiological role of ghrelin on GH regulation is age dependent.

In experimental animals, leptin stimulates GH secretion [28] and in clinical studies exogenous leptin failed to inhibit fasting-stimulated GH secretion [29]. The negative GH-leptin relationship observed in some studies probably reflects the known inverse correlation between total body fat and GH. It has been found after oral glucose that fasting and initial GH secretion and fasting leptin levels were higher in women than in men, suggesting that leptin does not participate in the sexual dimorphism of GH secretion after oral glucose [30]. In addition, treatment with recombinant growth hormone in patients with growth hormone deficiency decreased fat mass and leptin levels [31]. Taken together, all these data are in accordance with our results indicating that leptin does not participate in the decreased GH secretion in obesity. Insulin treatment inhibits GH release and reduces mRNA expression of GH, GHRH receptor and GHSR in primary cultures isolated from non-human primates [5]. In male mice, impaired pulsatile GH secretion occurs alongside progressive weight gain and thus precedes the development of obesity. In a similar way to our results in women, an inverse relationship between measures of pulsatile GH secretion and body weight, circulating levels of leptin and insulin has been demonstrated in mice [32]. Many indices in the study by Iranmanesh et al [23] point to insulin resistance (hyperinsulinemia) being involved in alterations in GH output, however, neither insulin concentrations nor HOMA-IR correlated with rebound GH secretion. Moreover, in the interesting study by Cornford et al, the reduction in GH secretion did not correlate with the increased insulin levels [8]. Other factors like sex steroids, GHRH, somatostatin, IGF-1, and IGFBP-1 could modulate ghrelin's control of GH secretion, as has been found in in healthy elderly men [33]. In addition, free fatty acids have been found to participate in GH hyposecretion in obesity [34].

The suppression of GH secretion in obesity may have important metabolic impact. We believe that this study employing multivariate analysis with both linear regression and generalized additive model strongly suggests that ghrelin secretion is an important peripheral regulator of GH secretion after oral glucose in healthy and obese women. The relationship between ghrelin, which increases food intake, and GH found during the late fed state would be beneficial to humans, as the anabolic changes induced by GH requires the presence of adequate nutrition [13, 25]. The findings of this study reveal that there is a significant correlation between ghrelin and GH secretion after OG in obese women, suggesting that the decreased ghrelin secretion in obesity is one of the mechanisms responsible for altered GH secretion in obesity. The recovery of the GH IGF-1 axis after weight loss suggests an acquired defect; however the decreased stimulation of GH release in obese subjects may promote the retention of fat mass, and contribute to perpetuation of the obese state. In premenopausal women with obesity, peak GH is inversely associated with intramyocellular and intrahepatic lipids. This suggests that low GH may contribute to insulin resistance in obesity through effects on muscle and intrahepatic lipids [35]. In the long-term, the functional GH deficiency could lead to a vicious circle, and to deterioration of the beta cell mass, and participating in the development of diabetes [36]. OG as a GH stimulus could have important clinical implications, as suggested by Iranmanesh et al [23]. This GH stimulus could be employed in clinical practice, and may provide a low risk means of evaluating endogenously regulated GH secretion without requiring the injection of insulin, GHRH, GHRP, or arginine in childhood, pregnancy or frail older adults.

Because our study was not interventional and the analysis is based on correlation, we cannot completely exclude the existence of one common or several separate factors that control both GH release and circulating ghrelin levels simultaneously. Therefore, a possible role for insulin as a common regulator of circulating ghrelin and GH after OG cannot be completely excluded, even though the correlation between GH and ghrelin persisted after adjusting for insulin. Another limitation is that we did not measure acylated ghrelin. Although acylated ghrelin has proved to be the biologically active form in the control of GH secretion, most of the leading studies on the correlation between GH and ghrelin secretion have focused on the estimation of total ghrelin [11, 12, 25, 37, 38] and there are concerns regarding the specificity of available acyl-ghrelin assays and, more importantly, the stability of plasma acylated ghrelin once collected [39]. In this sense, acylated ghrelin levels have been found normal or increased [40–42] but also decreased [43–45] in obesity. The present study has been done only in women, so the present results should not be extrapolated to men.

## Conclusions

These data suggest that ghrelin secretion is an important regulator of GH secretion after oral glucose in women, and that the decreased GH secretion of obesity after oral glucose is probably due to the altered ghrelin levels found in obese patients.
